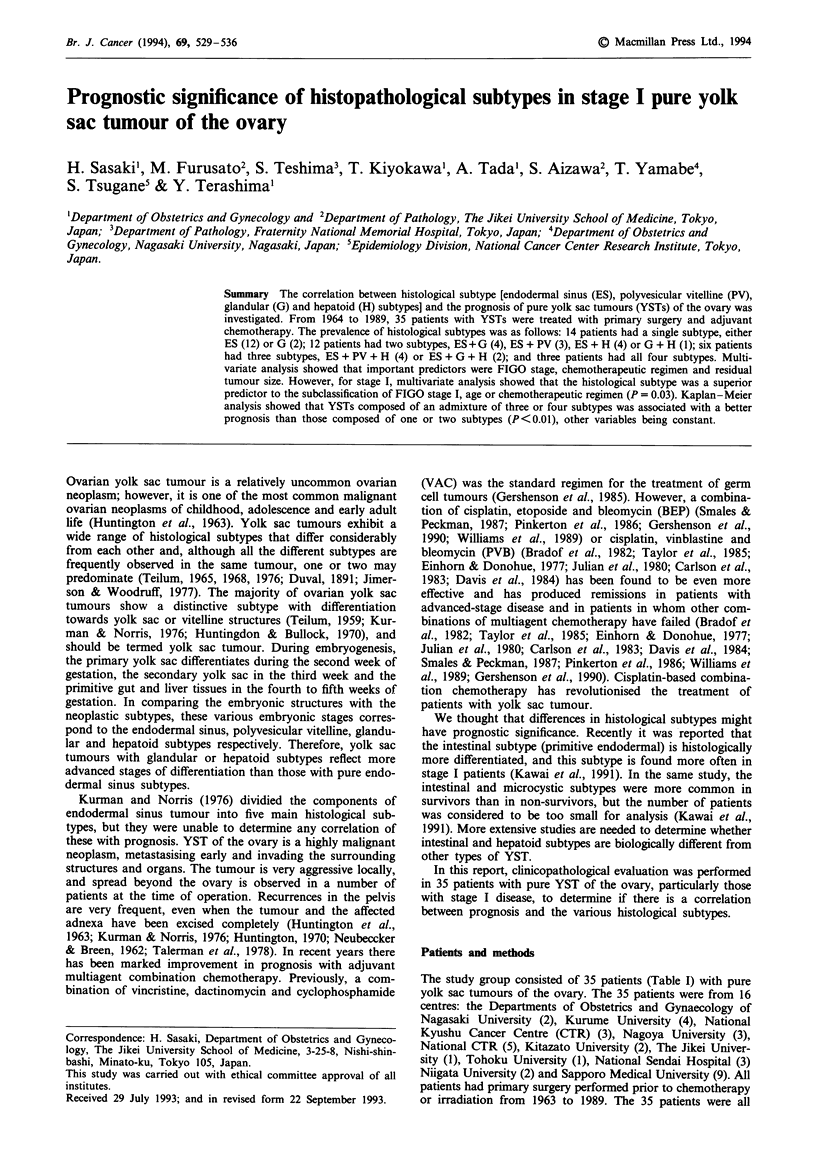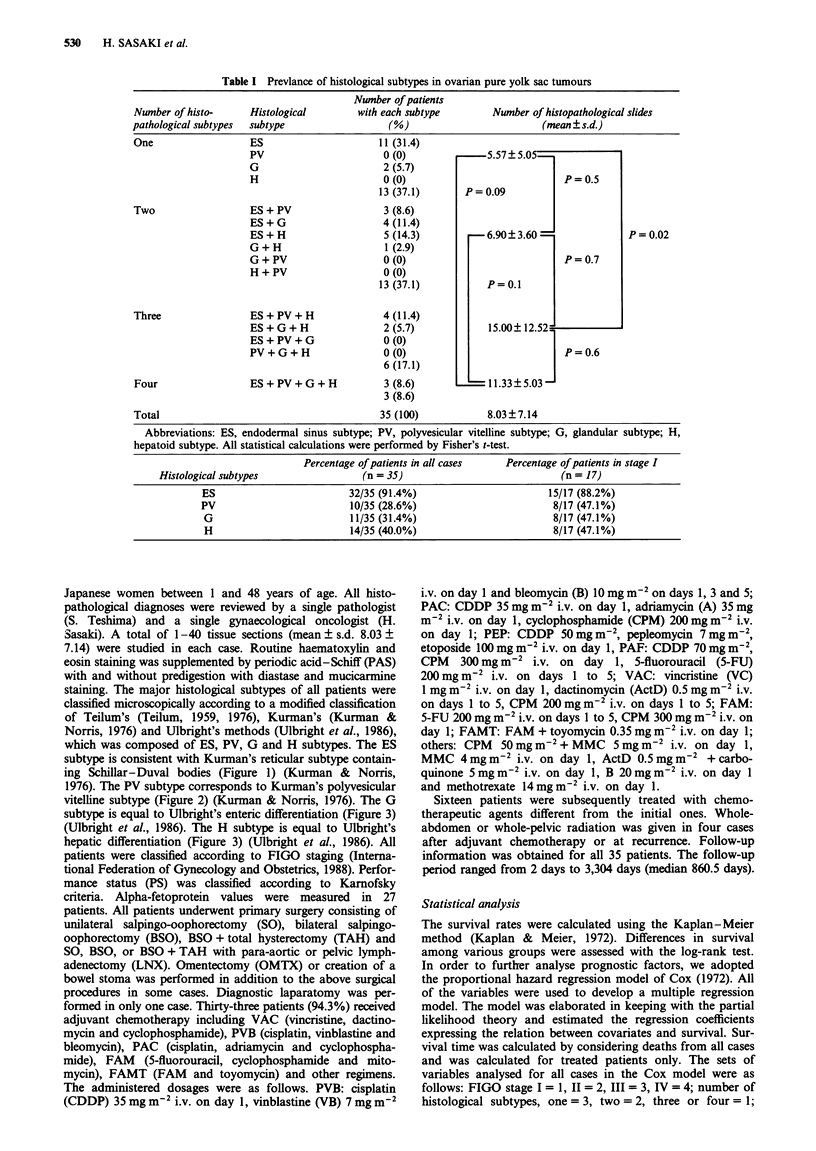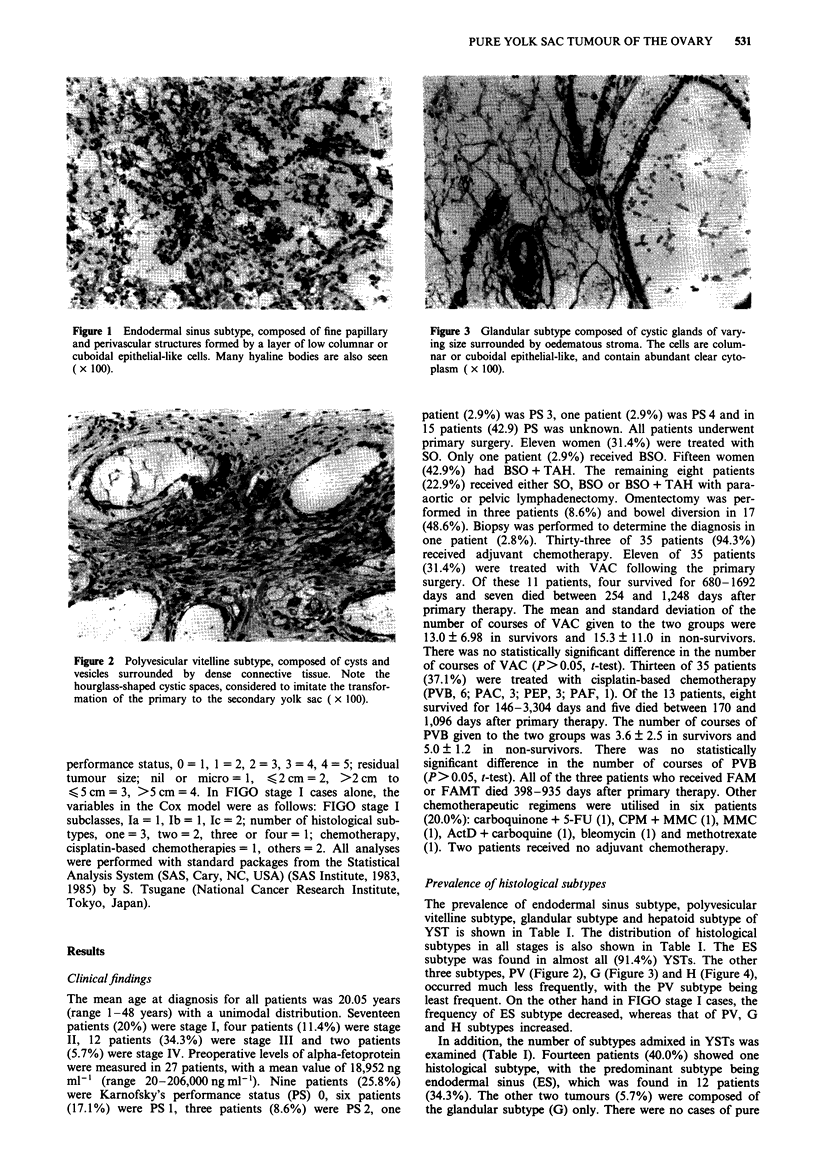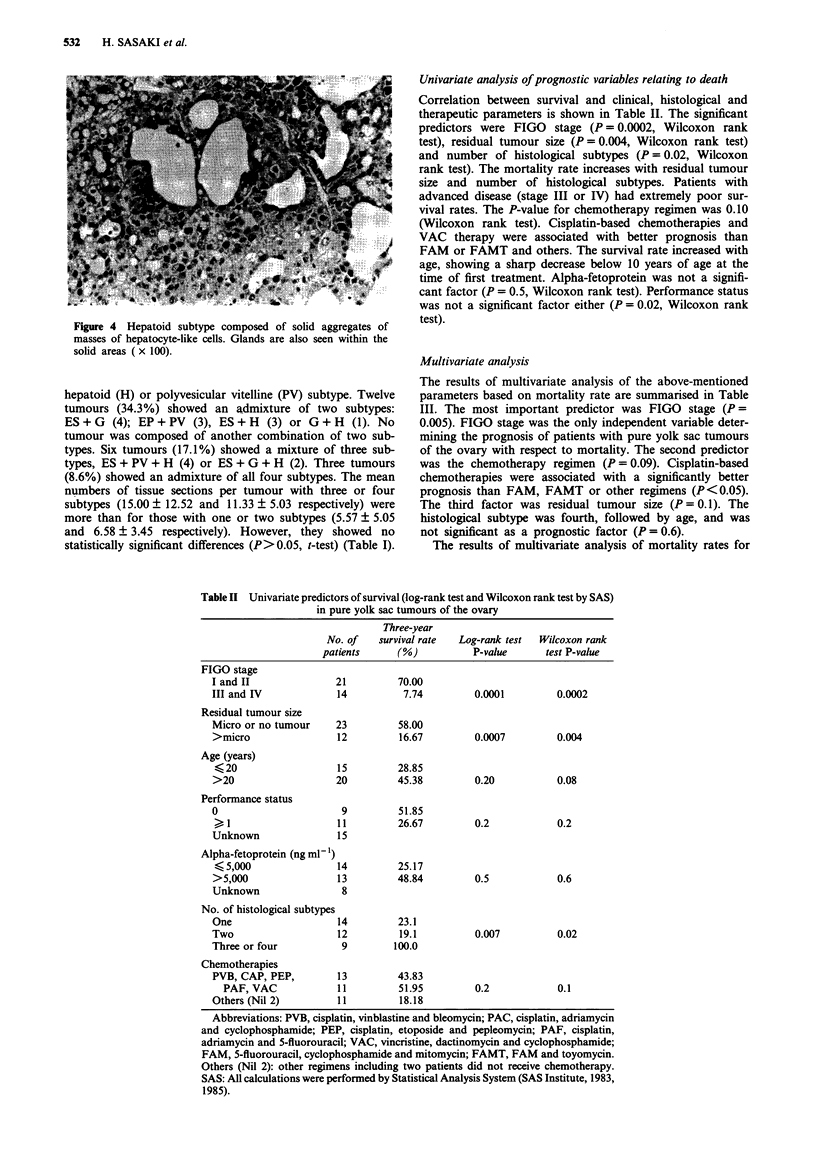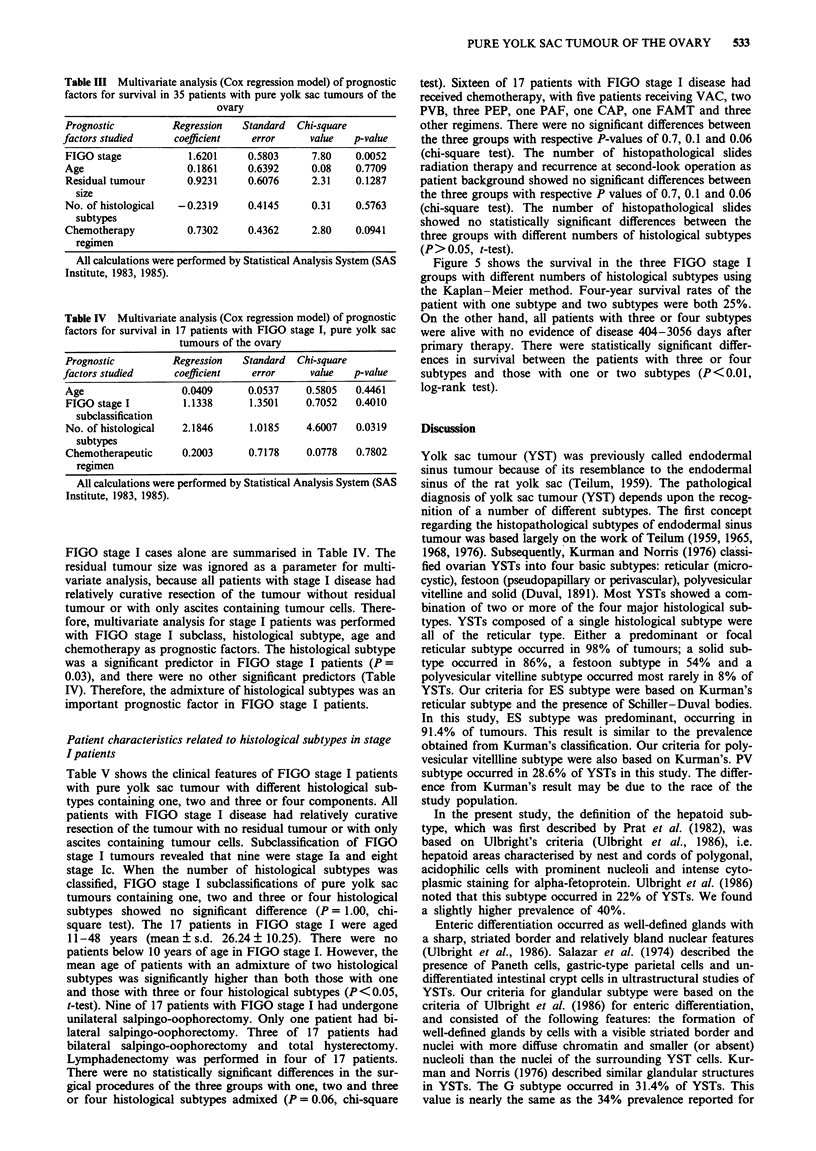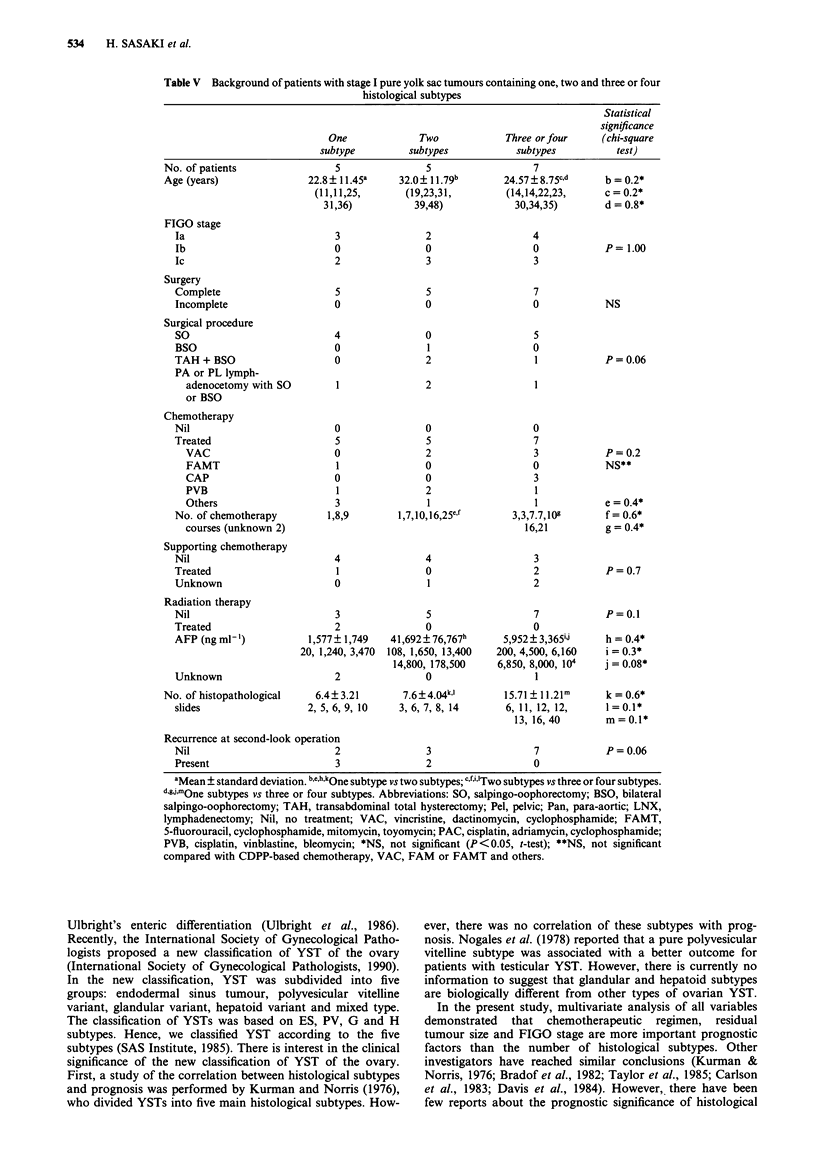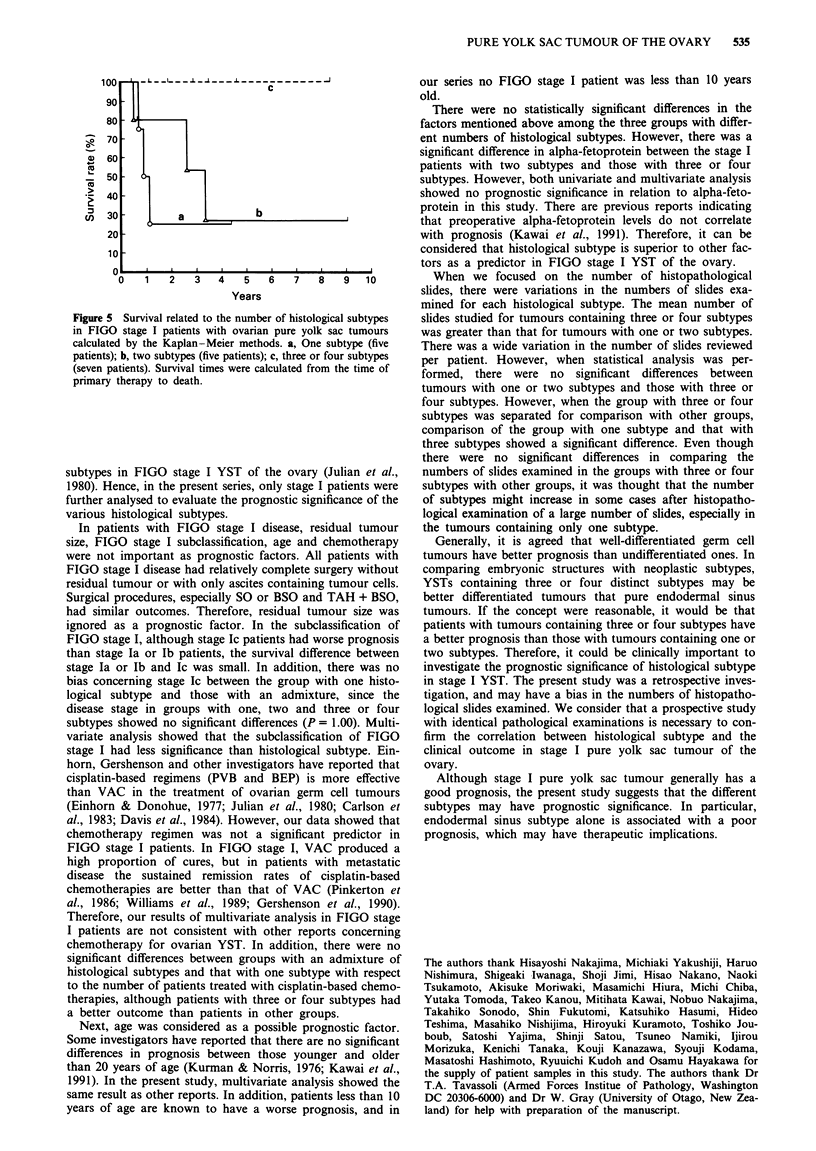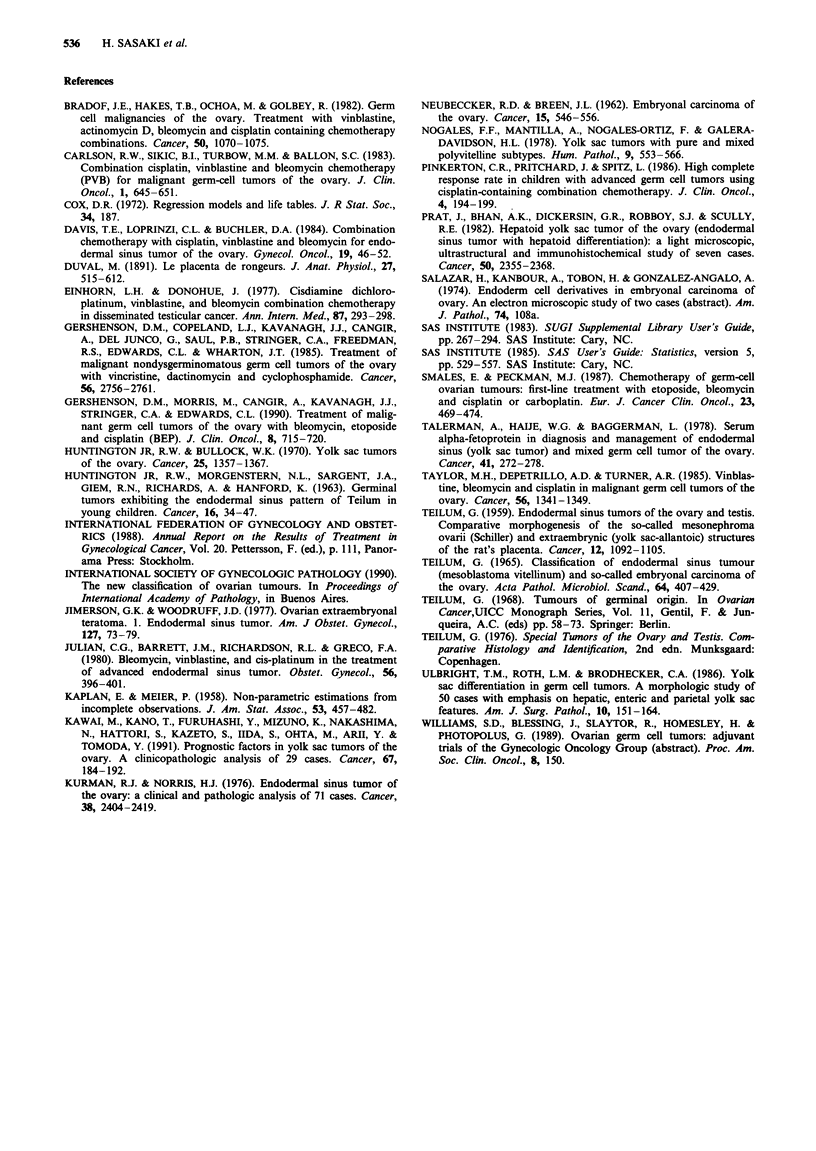# Prognostic significance of histopathological subtypes in stage I pure yolk sac tumour of the ovary.

**DOI:** 10.1038/bjc.1994.96

**Published:** 1994-03

**Authors:** H. Sasaki, M. Furusato, S. Teshima, T. Kiyokawa, A. Tada, S. Aizawa, T. Yamabe, S. Tsugane, Y. Terashima

**Affiliations:** Department of Obstetrics and Gynecology, Jikei University School of Medicine, Tokyo, Japan.

## Abstract

**Images:**


Br. J. Cancer (1994), 69, 529–536 © Macmillan Press Ltd., 1994

### Prognostic significance of histopathological subtypes in stage I pure yolk sac tumour of the ovary

H. Sasaki[1], M. Furusato[2], S. Teshima[3], T. Kiyokawa[1], A. Tada[1], S. Aizawa[2], T. Yamabe[4], S. Tsugane[5] & Y. Terashima[1]

[1]Department of Obstetrics and Gynecology and [2]Department of Pathology, The Jikei University School of Medicine, Tokyo, Japan; [3]Department of Pathology, Fraternity National Memorial Hospital, Tokyo, Japan; [4]Department of Obstetrics and Gynecology, Nagasaki University, Nagasaki, Japan; [5]Epidemiology Division, National Cancer Center Research Institute, Tokyo, Japan.

**Summary** The correlation between histological subtype [endodermal sinus (ES), polyvesicular vitelline (PV), glandular (G) and hepatoid (H) subtypes] and the prognosis of pure yolk sac tumours (YSTs) of the ovary was investigated. From 1964 to 1989, 35 patients with YSTs were treated with primary surgery and adjuvant chemotherapy. The prevalence of histological subtypes was as follows: 14 patients had a single subtype, either ES (12) or G (2); 12 patients had two subtypes, ES + G (4), ES + PV (3), ES + H (4) or G + H (1); six patients had three subtypes, ES + PV + H (4) or ES + G + H (2); and three patients had all four subtypes. Multivariate analysis showed that important predictors were FIGO stage, chemotherapeutic regimen and residual tumour size. However, for stage I, multivariate analysis showed that the histological subtype was a superior predictor to the subclassification of FIGO stage I, age or chemotherapeutic regimen ($P = 0.03$). Kaplan–Meier analysis showed that YSTs composed of an admixture of three or four subtypes was associated with a better prognosis than those composed of one or two subtypes ($P < 0.01$), other variables being constant.

Ovarian yolk sac tumour is a relatively uncommon ovarian neoplasm; however, it is one of the most common malignant ovarian neoplasms of childhood, adolescence and early adult life (Huntington *et al.*, 1963). Yolk sac tumours exhibit a wide range of histological subtypes that differ considerably from each other and, although all the different subtypes are frequently observed in the same tumour, one or two may predominate (Teilum, 1965, 1968, 1976; Duval, 1891; Jimerson & Woodruff, 1977). The majority of ovarian yolk sac tumours show a distinctive subtype with differentiation towards yolk sac or vitelline structures (Teilum, 1959; Kurman & Norris, 1976; Huntingdon & Bullock, 1970), and should be termed yolk sac tumour. During embryogenesis, the primary yolk sac differentiates during the second week of gestation, the secondary yolk sac in the third week and the primitive gut and liver tissues in the fourth to fifth weeks of gestation. In comparing the embryonic structures with the neoplastic subtypes, these various embryonic stages correspond to the endodermal sinus, polyvesicular vitelline, glandular and hepatoid subtypes respectively. Therefore, yolk sac tumours with glandular or hepatoid subtypes reflect more advanced stages of differentiation than those with pure endodermal sinus subtypes.

Kurman and Norris (1976) dividied the components of endodermal sinus tumour into five main histological subtypes, but they were unable to determine any correlation of these with prognosis. YST of the ovary is a highly malignant neoplasm, metastasising early and invading the surrounding structures and organs. The tumour is very aggressive locally, and spread beyond the ovary is observed in a number of patients at the time of operation. Recurrences in the pelvis are very frequent, even when the tumour and the affected adnexa have been excised completely (Huntington *et al.*, 1963; Kurman & Norris, 1976; Huntington, 1970; Neubeccker & Breen, 1962; Talerman *et al.*, 1978). In recent years there has been marked improvement in prognosis with adjuvant multiagent combination chemotherapy. Previously, a combination of vincristine, dactinomycin and cyclophosphamide (VAC) was the standard regimen for the treatment of germ cell tumours (Gershenson *et al.*, 1985). However, a combination of cisplatin, etoposide and bleomycin (BEP) (Smales & Peckman, 1987; Pinkerton *et al.*, 1986; Gershenson *et al.*, 1990; Williams *et al.*, 1989) or cisplatin, vinblastine and bleomycin (PVB) (Bradof *et al.*, 1982; Taylor *et al.*, 1985; Einhorn & Donohue, 1977; Julian *et al.*, 1980; Carlson *et al.*, 1983; Davis *et al.*, 1984) has been found to be even more effective and has produced remissions in patients with advanced-stage disease and in patients in whom other combinations of multiagent chemotherapy have failed (Bradof *et al.*, 1982; Taylor *et al.*, 1985; Einhorn & Donohue, 1977; Julian *et al.*, 1980; Carlson *et al.*, 1983; Davis *et al.*, 1984; Smales & Peckman, 1987; Pinkerton *et al.*, 1986; Williams *et al.*, 1989; Gershenson *et al.*, 1990). Cisplatin-based combination chemotherapy has revolutionised the treatment of patients with yolk sac tumour.

We thought that differences in histological subtypes might have prognostic significance. Recently it was reported that the intestinal subtype (primitive endodermal) is histologically more differentiated, and this subtype is found more often in stage I patients (Kawai *et al.*, 1991). In the same study, the intestinal and microcystic subtypes were more common in survivors than in non-survivors, but the number of patients was considered to be too small for analysis (Kawai *et al.*, 1991). More extensive studies are needed to determine whether intestinal and hepatoid subtypes are biologically different from other types of YST.

In this report, clinicopathological evaluation was performed in 35 patients with pure YST of the ovary, particularly those with stage I disease, to determine if there is a correlation between prognosis and the various histological subtypes.

### Patients and methods

The study group consisted of 35 patients (Table I) with pure yolk sac tumours of the ovary. The 35 patients were from 16 centres: the Departments of Obstetrics and Gynaecology of Nagasaki University (2), Kurume University (4), National Kyushu Cancer Centre (CTR) (3), Nagoya University (3), National CTR (5), Kitazato University (2), The Jikei University (1), Tohoku University (1), National Sendai Hospital (3) Niigata University (2) and Sapporo Medical University (9). All patients had primary surgery performed prior to chemotherapy or irradiation from 1963 to 1989. The 35 patients were all

Correspondence: H. Sasaki, Department of Obstetrics and Gynecology, The Jikei University School of Medicine, 3-25-8, Nishi-shinbashi, Minato-ku, Tokyo 105, Japan.

This study was carried out with ethical committee approval of all institutes.

Received 29 July 1993; and in revised form 22 September 1993.

**Table I** Prevlance of histological subtypes in ovarian pure yolk sac tumours

| *Number of histo-pathological subtypes* | *Histological subtype* | *Number of patients with each subtype (%)* | *Number of histopathological slides (mean ± s.d.)* | |
|---|---|---|---|---|
| One | ES | 11 (31.4) | | |
| | PV | 0 (0) | 5.57 ± 5.05 | |
| | G | 2 (5.7) | | |
| | H | 0 (0) | | $P = 0.5$ |
| | | 13 (37.1) | $P = 0.09$ | |
| Two | ES + PV | 3 (8.6) | | |
| | ES + G | 4 (11.4) | | |
| | ES + H | 5 (14.3) | 6.90 ± 3.60 | $P = 0.02$ |
| | G + H | 1 (2.9) | | |
| | G + PV | 0 (0) | | $P = 0.7$ |
| | H + PV | 0 (0) | | |
| | | 13 (37.1) | $P = 0.1$ | |
| Three | ES + PV + H | 4 (11.4) | | |
| | ES + G + H | 2 (5.7) | 15.00 ± 12.52 | |
| | ES + PV + G | 0 (0) | | |
| | PV + G + H | 0 (0) | | $P = 0.6$ |
| | | 6 (17.1) | | |
| Four | ES + PV + G + H | 3 (8.6) | 11.33 ± 5.03 | |
| | | 3 (8.6) | | |
| Total | | 35 (100) | 8.03 ± 7.14 | |

Abbreviations: ES, endodermal sinus subtype; PV, polyvesicular vitelline subtype; G, glandular subtype; H, hepatoid subtype. All statistical calculations were performed by Fisher's *t*-test.

| *Histological subtypes* | *Percentage of patients in all cases (n = 35)* | *Percentage of patients in stage I (n = 17)* |
|---|---|---|
| ES | 32/35 (91.4%) | 15/17 (88.2%) |
| PV | 10/35 (28.6%) | 8/17 (47.1%) |
| G | 11/35 (31.4%) | 8/17 (47.1%) |
| H | 14/35 (40.0%) | 8/17 (47.1%) |

Japanese women between 1 and 48 years of age. All histopathological diagnoses were reviewed by a single pathologist (S. Teshima) and a single gynaecological oncologist (H. Sasaki). A total of 1–40 tissue sections (mean ± s.d. 8.03 ± 7.14) were studied in each case. Routine haematoxylin and eosin staining was supplemented by periodic acid–Schiff (PAS) with and without predigestion with diastase and mucicarmine staining. The major histological subtypes of all patients were classified microscopically according to a modified classification of Teilum's (Teilum, 1959, 1976), Kurman's (Kurman & Norris, 1976) and Ulbright's methods (Ulbright *et al.*, 1986), which was composed of ES, PV, G and H subtypes. The ES subtype is consistent with Kurman's reticular subtype containing Schillar–Duval bodies (Figure 1) (Kurman & Norris, 1976). The PV subtype corresponds to Kurman's polyvesicular vitelline subtype (Figure 2) (Kurman & Norris, 1976). The G subtype is equal to Ulbright's enteric differentiation (Figure 3) (Ulbright *et al.*, 1986). The H subtype is equal to Ulbright's hepatic differentiation (Figure 3) (Ulbright *et al.*, 1986). All patients were classified according to FIGO staging (International Federation of Gynecology and Obstetrics, 1988). Performance status (PS) was classified according to Karnofsky criteria. Alpha-fetoprotein values were measured in 27 patients. All patients underwent primary surgery consisting of unilateral salpingo-oophorectomy (SO), bilateral salpingo-oophorectomy (BSO), BSO + total hysterectomy (TAH) and SO, BSO, or BSO + TAH with para-aortic or pelvic lymphadenectomy (LNX). Omentectomy (OMTX) or creation of a bowel stoma was performed in addition to the above surgical procedures in some cases. Diagnostic laparatomy was performed in only one case. Thirty-three patients (94.3%) received adjuvant chemotherapy including VAC (vincristine, dactinomycin and cyclophosphamide), PVB (cisplatin, vinblastine and bleomycin), PAC (cisplatin, adriamycin and cyclophosphamide), FAM (5-fluorouracil, cyclophosphamide and mitomycin), FAMT (FAM and toyomycin) and other regimens. The administered dosages were as follows. PVB: cisplatin (CDDP) 35 mg $m^{-2}$ i.v. on day 1, vinblastine (VB) 7 mg $m^{-2}$ i.v. on day 1 and bleomycin (B) 10 mg $m^{-2}$ on days 1, 3 and 5; PAC: CDDP 35 mg $m^{-2}$ i.v. on day 1, adriamycin (A) 35 mg $m^{-2}$ i.v. on day 1, cyclophosphamide (CPM) 200 mg $m^{-2}$ i.v. on day 1; PEP: CDDP 50 mg $m^{-2}$, pepleomycin 7 mg $m^{-2}$, etoposide 100 mg $m^{-2}$ i.v. on day 1, PAF: CDDP 70 mg $m^{-2}$, CPM 300 mg $m^{-2}$ i.v. on day 1, 5-fluorouracil (5-FU) 200 mg $m^{-2}$ i.v. on days 1 to 5; VAC: vincristine (VC) 1 mg $m^{-2}$ i.v. on day 1, dactinomycin (ActD) 0.5 mg $m^{-2}$ i.v. on days 1 to 5, CPM 200 mg $m^{-2}$ i.v. on days 1 to 5; FAM: 5-FU 200 mg $m^{-2}$ i.v. on days 1 to 5, CPM 300 mg $m^{-2}$ i.v. on day 1; FAMT: FAM + toyomycin 0.35 mg $m^{-2}$ i.v. on day 1; others: CPM 50 mg $m^{-2}$ + MMC 5 mg $m^{-2}$ i.v. on day 1, MMC 4 mg $m^{-2}$ i.v. on day 1, ActD 0.5 mg $m^{-2}$ + carboquinone 5 mg $m^{-2}$ i.v. on day 1, B 20 mg $m^{-2}$ i.v. on day 1 and methotrexate 14 mg $m^{-2}$ i.v. on day 1.

Sixteen patients were subsequently treated with chemotherapeutic agents different from the initial ones. Whole-abdomen or whole-pelvic radiation was given in four cases after adjuvant chemotherapy or at recurrence. Follow-up information was obtained for all 35 patients. The follow-up period ranged from 2 days to 3,304 days (median 860.5 days).

### *Statistical analysis*

The survival rates were calculated using the Kaplan–Meier method (Kaplan & Meier, 1972). Differences in survival among various groups were assessed with the log-rank test. In order to further analyse prognostic factors, we adopted the proportional hazard regression model of Cox (1972). All of the variables were used to develop a multiple regression model. The model was elaborated in keeping with the partial likelihood theory and estimated the regression coefficients expressing the relation between covariates and survival. Survival time was calculated by considering deaths from all cases and was calculated for treated patients only. The sets of variables analysed for all cases in the Cox model were as follows: FIGO stage I = 1, II = 2, III = 3, IV = 4; number of histological subtypes, one = 3, two = 2, three or four = 1;

**Figure 1** Endodermal sinus subtype, composed of fine papillary and perivascular structures formed by a layer of low columnar or cuboidal epithelial-like cells. Many hyaline bodies are also seen ( × 100).

**Figure 3** Glandular subtype composed of cystic glands of varying size surrounded by oedematous stroma. The cells are columnar or cuboidal epithelial-like, and contain abundant clear cytoplasm ( × 100).

**Figure 2** Polyvesicular vitelline subtype, composed of cysts and vesicles surrounded by dense connective tissue. Note the hourglass-shaped cystic spaces, considered to imitate the transformation of the primary to the secondary yolk sac ( × 100).

performance status, 0 = 1, 1 = 2, 2 = 3, 3 = 4, 4 = 5; residual tumour size; nil or micro = 1, $\leqslant 2$ cm = 2, $>2$ cm to $\leqslant 5$ cm = 3, $>5$ cm = 4. In FIGO stage I cases alone, the variables in the Cox model were as follows: FIGO stage I subclasses, Ia = 1, Ib = 1, Ic = 2; number of histological subtypes, one = 3, two = 2, three or four = 1; chemotherapy, cisplatin-based chemotherapies = 1, others = 2. All analyses were performed with standard packages from the Statistical Analysis System (SAS, Cary, NC, USA) (SAS Institute, 1983, 1985) by S. Tsugane (National Cancer Research Institute, Tokyo, Japan).

### Results

### *Clinical findings*

The mean age at diagnosis for all patients was 20.05 years (range 1–48 years) with a unimodal distribution. Seventeen patients (20%) were stage I, four patients (11.4%) were stage II, 12 patients (34.3%) were stage III and two patients (5.7%) were stage IV. Preoperative levels of alpha-fetoprotein were measured in 27 patients, with a mean value of 18,952 ng $ml^{-1}$ (range 20–206,000 ng $ml^{-1}$). Nine patients (25.8%) were Karnofsky's performance status (PS) 0, six patients (17.1%) were PS 1, three patients (8.6%) were PS 2, one patient (2.9%) was PS 3, one patient (2.9%) was PS 4 and in 15 patients (42.9) PS was unknown. All patients underwent primary surgery. Eleven women (31.4%) were treated with SO. Only one patient (2.9%) received BSO. Fifteen women (42.9%) had BSO + TAH. The remaining eight patients (22.9%) received either SO, BSO or BSO + TAH with para-aortic or pelvic lymphadenectomy. Omentectomy was performed in three patients (8.6%) and bowel diversion in 17 (48.6%). Biopsy was performed to determine the diagnosis in one patient (2.8%). Thirty-three of 35 patients (94.3%) received adjuvant chemotherapy. Eleven of 35 patients (31.4%) were treated with VAC following the primary surgery. Of these 11 patients, four survived for 680–1692 days and seven died between 254 and 1,248 days after primary therapy. The mean and standard deviation of the number of courses of VAC given to the two groups were $13.0 \pm 6.98$ in survivors and $15.3 \pm 11.0$ in non-survivors. There was no statistically significant difference in the number of courses of VAC ($P > 0.05$, *t*-test). Thirteen of 35 patients (37.1%) were treated with cisplatin-based chemotherapy (PVB, 6; PAC, 3; PEP, 3; PAF, 1). Of the 13 patients, eight survived for 146–3,304 days and five died between 170 and 1,096 days after primary therapy. The number of courses of PVB given to the two groups was $3.6 \pm 2.5$ in survivors and $5.0 \pm 1.2$ in non-survivors. There was no statistically significant difference in the number of courses of PVB ($P > 0.05$, *t*-test). All of the three patients who received FAM or FAMT died 398–935 days after primary therapy. Other chemotherapeutic regimens were utilised in six patients (20.0%): carboquinone + 5-FU (1), CPM + MMC (1), MMC (1), ActD + carboquine (1), bleomycin (1) and methotrexate (1). Two patients received no adjuvant chemotherapy.

### *Prevalence of histological subtypes*

The prevalence of endodermal sinus subtype, polyvesicular vitelline subtype, glandular subtype and hepatoid subtype of YST is shown in Table I. The distribution of histological subtypes in all stages is also shown in Table I. The ES subtype was found in almost all (91.4%) YSTs. The other three subtypes, PV (Figure 2), G (Figure 3) and H (Figure 4), occurred much less frequently, with the PV subtype being least frequent. On the other hand in FIGO stage I cases, the frequency of ES subtype decreased, whereas that of PV, G and H subtypes increased.

In addition, the number of subtypes admixed in YSTs was examined (Table I). Fourteen patients (40.0%) showed one histological subtype, with the predominant subtype being endodermal sinus (ES), which was found in 12 patients (34.3%). The other two tumours (5.7%) were composed of the glandular subtype (G) only. There were no cases of pure

**Figure 4** Hepatoid subtype composed of solid aggregates of masses of hepatocyte-like cells. Glands are also seen within the solid areas ( × 100).

hepatoid (H) or polyvesicular vitelline (PV) subtype. Twelve tumours (34.3%) showed an admixture of two subtypes: ES + G (4); EP + PV (3), ES + H (3) or G + H (1). No tumour was composed of another combination of two subtypes. Six tumours (17.1%) showed a mixture of three subtypes, ES + PV + H (4) or ES + G + H (2). Three tumours (8.6%) showed an admixture of all four subtypes. The mean numbers of tissue sections per tumour with three or four subtypes (15.00 ± 12.52 and 11.33 ± 5.03 respectively) were more than for those with one or two subtypes (5.57 ± 5.05 and 6.58 ± 3.45 respectively). However, they showed no statistically significant differences ($P > 0.05$, $t$-test) (Table I).

*Univariate analysis of prognostic variables relating to death*

Correlation between survival and clinical, histological and therapeutic parameters is shown in Table II. The significant predictors were FIGO stage ($P = 0.0002$, Wilcoxon rank test), residual tumour size ($P = 0.004$, Wilcoxon rank test) and number of histological subtypes ($P = 0.02$, Wilcoxon rank test). The mortality rate increases with residual tumour size and number of histological subtypes. Patients with advanced disease (stage III or IV) had extremely poor survival rates. The $P$-value for chemotherapy regimen was 0.10 (Wilcoxon rank test). Cisplatin-based chemotherapies and VAC therapy were associated with better prognosis than FAM or FAMT and others. The survival rate increased with age, showing a sharp decrease below 10 years of age at the time of first treatment. Alpha-fetoprotein was not a significant factor ($P = 0.5$, Wilcoxon rank test). Performance status was not a significant factor either ($P = 0.02$, Wilcoxon rank test).

*Multivariate analysis*

The results of multivariate analysis of the above-mentioned parameters based on mortality rate are summarised in Table III. The most important predictor was FIGO stage ($P = 0.005$). FIGO stage was the only independent variable determining the prognosis of patients with pure yolk sac tumours of the ovary with respect to mortality. The second predictor was the chemotherapy regimen ($P = 0.09$). Cisplatin-based chemotherapies were associated with a significantly better prognosis than FAM, FAMT or other regimens ($P < 0.05$). The third factor was residual tumour size ($P = 0.1$). The histological subtype was fourth, followed by age, and was not significant as a prognostic factor ($P = 0.6$).

The results of multivariate analysis of mortality rates for

**Table II** Univariate predictors of survival (log-rank test and Wilcoxon rank test by SAS) in pure yolk sac tumours of the ovary

| | *No. of patients* | *Three-year survival rate (%)* | *Log-rank test P-value* | *Wilcoxon rank test P-value* |
|---|---|---|---|---|
| FIGO stage | | | | |
| I and II | 21 | 70.00 | | |
| III and IV | 14 | 7.74 | 0.0001 | 0.0002 |
| Residual tumour size | | | | |
| Micro or no tumour | 23 | 58.00 | | |
| >micro | 12 | 16.67 | 0.0007 | 0.004 |
| Age (years) | | | | |
| ⩽20 | 15 | 28.85 | | |
| >20 | 20 | 45.38 | 0.20 | 0.08 |
| Performance status | | | | |
| 0 | 9 | 51.85 | | |
| ⩾1 | 11 | 26.67 | 0.2 | 0.2 |
| Unknown | 15 | | | |
| Alpha-fetoprotein (ng $ml^{-1}$) | | | | |
| ⩽5,000 | 14 | 25.17 | | |
| >5,000 | 13 | 48.84 | 0.5 | 0.6 |
| Unknown | 8 | | | |
| No. of histological subtypes | | | | |
| One | 14 | 23.1 | | |
| Two | 12 | 19.1 | 0.007 | 0.02 |
| Three or four | 9 | 100.0 | | |
| Chemotherapies | | | | |
| PVB, CAP, PEP, | 13 | 43.83 | | |
| PAF, VAC | 11 | 51.95 | 0.2 | 0.1 |
| Others (Nil 2) | 11 | 18.18 | | |

Abbreviations: PVB, cisplatin, vinblastine and bleomycin; PAC, cisplatin, adriamycin and cyclophosphamide; PEP, cisplatin, etoposide and pepleomycin; PAF, cisplatin, adriamycin and 5-fluorouracil; VAC, vincristine, dactinomycin and cyclophosphamide; FAM, 5-fluorouracil, cyclophosphamide and mitomycin; FAMT, FAM and toyomycin. Others (Nil 2): other regimens including two patients did not receive chemotherapy. SAS: All calculations were performed by Statistical Analysis System (SAS Institute, 1983, 1985).

**Table III** Multivariate analysis (Cox regression model) of prognostic factors for survival in 35 patients with pure yolk sac tumours of the ovary

| *Prognostic factors studied* | *Regression coefficient* | *Standard error* | *Chi-square value* | *p-value* |
|---|---|---|---|---|
| FIGO stage | 1.6201 | 0.5803 | 7.80 | 0.0052 |
| Age | 0.1861 | 0.6392 | 0.08 | 0.7709 |
| Residual tumour size | 0.9231 | 0.6076 | 2.31 | 0.1287 |
| No. of histological subtypes | −0.2319 | 0.4145 | 0.31 | 0.5763 |
| Chemotherapy regimen | 0.7302 | 0.4362 | 2.80 | 0.0941 |

All calculations were performed by Statistical Analysis System (SAS Institute, 1983, 1985).

**Table IV** Multivariate analysis (Cox regression model) of prognostic factors for survival in 17 patients with FIGO stage I, pure yolk sac tumours of the ovary

| *Prognostic factors studied* | *Regression coefficient* | *Standard error* | *Chi-square value* | *p-value* |
|---|---|---|---|---|
| Age | 0.0409 | 0.0537 | 0.5805 | 0.4461 |
| FIGO stage I subclassification | 1.1338 | 1.3501 | 0.7052 | 0.4010 |
| No. of histological subtypes | 2.1846 | 1.0185 | 4.6007 | 0.0319 |
| Chemotherapeutic regimen | 0.2003 | 0.7178 | 0.0778 | 0.7802 |

All calculations were performed by Statistical Analysis System (SAS Institute, 1983, 1985).

FIGO stage I cases alone are summarised in Table IV. The residual tumour size was ignored as a parameter for multivariate analysis, because all patients with stage I disease had relatively curative resection of the tumour without residual tumour or with only ascites containing tumour cells. Therefore, multivariate analysis for stage I patients was performed with FIGO stage I subclass, histological subtype, age and chemotherapy as prognostic factors. The histological subtype was a significant predictor in FIGO stage I patients ($P = 0.03$), and there were no other significant predictors (Table IV). Therefore, the admixture of histological subtypes was an important prognostic factor in FIGO stage I patients.

### *Patient characteristics related to histological subtypes in stage I patients*

Table V shows the clinical features of FIGO stage I patients with pure yolk sac tumour with different histological subtypes containing one, two and three or four components. All patients with FIGO stage I disease had relatively curative resection of the tumour with no residual tumour or with only ascites containing tumour cells. Subclassification of FIGO stage I tumours revealed that nine were stage Ia and eight stage Ic. When the number of histological subtypes was classified, FIGO stage I subclassifications of pure yolk sac tumours containing one, two and three or four histological subtypes showed no significant difference ($P = 1.00$, chi-square test). The 17 patients in FIGO stage I were aged 11–48 years (mean ± s.d. 26.24 ± 10.25). There were no patients below 10 years of age in FIGO stage I. However, the mean age of patients with an admixture of two histological subtypes was significantly higher than both those with one and those with three or four histological subtypes ($P < 0.05$, *t*-test). Nine of 17 patients with FIGO stage I had undergone unilateral salpingo-oophorectomy. Only one patient had bilateral salpingo-oophorectomy. Three of 17 patients had bilateral salpingo-oophorectomy and total hysterectomy. Lymphadenectomy was performed in four of 17 patients. There were no statistically significant differences in the surgical procedures of the three groups with one, two and three or four histological subtypes admixed ($P = 0.06$, chi-square test). Sixteen of 17 patients with FIGO stage I disease had received chemotherapy, with five patients receiving VAC, two PVB, three PEP, one PAF, one CAP, one FAMT and three other regimens. There were no significant differences between the three groups with respective *P*-values of 0.7, 0.1 and 0.06 (chi-square test). The number of histopathological slides radiation therapy and recurrence at second-look operation as patient background showed no significant differences between the three groups with respective *P* values of 0.7, 0.1 and 0.06 (chi-square test). The number of histopathological slides showed no statistically significant differences between the three groups with different numbers of histological subtypes ($P > 0.05$, *t*-test).

Figure 5 shows the survival in the three FIGO stage I groups with different numbers of histological subtypes using the Kaplan–Meier method. Four-year survival rates of the patient with one subtype and two subtypes were both 25%. On the other hand, all patients with three or four subtypes were alive with no evidence of disease 404–3056 days after primary therapy. There were statistically significant differences in survival between the patients with three or four subtypes and those with one or two subtypes ($P < 0.01$, log-rank test).

### Discussion

Yolk sac tumour (YST) was previously called endodermal sinus tumour because of its resemblance to the endodermal sinus of the rat yolk sac (Teilum, 1959). The pathological diagnosis of yolk sac tumour (YST) depends upon the recognition of a number of different subtypes. The first concept regarding the histopathological subtypes of endodermal sinus tumour was based largely on the work of Teilum (1959, 1965, 1968, 1976). Subsequently, Kurman and Norris (1976) classified ovarian YSTs into four basic subtypes: reticular (microcystic), festoon (pseudopapillary or perivascular), polyvesicular vitelline and solid (Duval, 1891). Most YSTs showed a combination of two or more of the four major histological subtypes. YSTs composed of a single histological subtype were all of the reticular type. Either a predominant or focal reticular subtype occurred in 98% of tumours; a solid subtype occurred in 86%, a festoon subtype in 54% and a polyvesicular vitelline subtype occurred most rarely in 8% of YSTs. Our criteria for ES subtype were based on Kurman's reticular subtype and the presence of Schiller–Duval bodies. In this study, ES subtype was predominant, occurring in 91.4% of tumours. This result is similar to the prevalence obtained from Kurman's classification. Our criteria for polyvesicular vitellline subtype were also based on Kurman's. PV subtype occurred in 28.6% of YSTs in this study. The difference from Kurman's result may be due to the race of the study population.

In the present study, the definition of the hepatoid subtype, which was first described by Prat *et al.* (1982), was based on Ulbright's criteria (Ulbright *et al.*, 1986), i.e. hepatoid areas characterised by nest and cords of polygonal, acidophilic cells with prominent nucleoli and intense cytoplasmic staining for alpha-fetoprotein. Ulbright *et al.* (1986) noted that this subtype occurred in 22% of YSTs. We found a slightly higher prevalence of 40%.

Enteric differentiation occurred as well-defined glands with a sharp, striated border and relatively bland nuclear features (Ulbright *et al.*, 1986). Salazar *et al.* (1974) described the presence of Paneth cells, gastric-type parietal cells and undifferentiated intestinal crypt cells in ultrastructural studies of YSTs. Our criteria for glandular subtype were based on the criteria of Ulbright *et al.* (1986) for enteric differentiation, and consisted of the following features: the formation of well-defined glands by cells with a visible striated border and nuclei with more diffuse chromatin and smaller (or absent) nucleoli than the nuclei of the surrounding YST cells. Kurman and Norris (1976) described similar glandular structures in YSTs. The G subtype occurred in 31.4% of YSTs. This value is nearly the same as the 34% prevalence reported for

**Table V** Background of patients with stage I pure yolk sac tumours containing one, two and three or four histological subtypes

| | *One subtype* | *Two subtypes* | *Three or four subtypes* | *Statistical significance (chi-square test)* |
|---|---|---|---|---|
| No. of patients | 5 | 5 | 7 | |
| Age (years) | 22.8 ± 11.45[a] (11,11,25, 31,36) | 32.0 ± 11.79[b] (19,23,31, 39,48) | 24.57 ± 8.75[c,d] (14,14,22,23, 30,34,35) | b = 0.2* c = 0.2* d = 0.8* |
| FIGO stage | | | | |
| Ia | 3 | 2 | 4 | |
| Ib | 0 | 0 | 0 | $P = 1.00$ |
| Ic | 2 | 3 | 3 | |
| Surgery | | | | |
| Complete | 5 | 5 | 7 | |
| Incomplete | 0 | 0 | 0 | NS |
| Surgical procedure | | | | |
| SO | 4 | 0 | 5 | |
| BSO | 0 | 1 | 0 | |
| TAH + BSO | 0 | 2 | 1 | $P = 0.06$ |
| PA or PL lymph-adenocetomy with SO or BSO | 1 | 2 | 1 | |
| Chemotherapy | | | | |
| Nil | 0 | 0 | 0 | |
| Treated | 5 | 5 | 7 | |
| VAC | 0 | 2 | 3 | $P = 0.2$ |
| FAMT | 1 | 0 | 0 | NS** |
| CAP | 0 | 0 | 3 | |
| PVB | 1 | 2 | 1 | |
| Others | 3 | 1 | 1 | e = 0.4* |
| No. of chemotherapy courses (unknown 2) | 1,8,9 | 1,7,10,16,25[e,f] | 3,3,7.7,10[g] 16,21 | f = 0.6* g = 0.4* |
| Supporting chemotherapy | | | | |
| Nil | 4 | 4 | 3 | |
| Treated | 1 | 0 | 2 | $P = 0.7$ |
| Unknown | 0 | 1 | 2 | |
| Radiation therapy | | | | |
| Nil | 3 | 5 | 7 | $P = 0.1$ |
| Treated | 2 | 0 | 0 | |
| AFP (ng $ml^{-1}$) | 1,577 ± 1,749 20, 1,240, 3,470 | 41,692 ± 76,767[h] 108, 1,650, 13,400 14,800, 178,500 | 5,952 ± 3,365[i,j] 200, 4,500, 6,160 6,850, 8,000, $10^4$ | h = 0.4* i = 0.3* j = 0.08* |
| Unknown | 2 | 0 | 1 | |
| No. of histopathological slides | 6.4 ± 3.21 2, 5, 6, 9, 10 | 7.6 ± 4.04[k,l] 3, 6, 7, 8, 14 | 15.71 ± 11.21[m] 6, 11, 12, 12, 13, 16, 40 | k = 0.6* l = 0.1* m = 0.1* |
| Recurrence at second-look operation | | | | |
| Nil | 2 | 3 | 7 | $P = 0.06$ |
| Present | 3 | 2 | 0 | |

[a]Mean ± standard deviation. [b,e,h,k]One subtype *vs* two subtypes; [c,f,i,l]Two subtypes *vs* three or four subtypes. [d,g,j,m]One subtypes *vs* three or four subtypes. Abbreviations: SO, salpingo-oophorectomy; BSO, bilateral salpingo-oophorectomy; TAH, transabdominal total hysterectomy; Pel, pelvic; Pan, para-aortic; LNX, lymphadenectomy; Nil, no treatment; VAC, vincristine, dactinomycin, cyclophosphamide; FAMT, 5-fluorouracil, cyclophosphamide, mitomycin, toyomycin; PAC, cisplatin, adriamycin, cyclophosphamide; PVB, cisplatin, vinblastine, bleomycin; *NS, not significant ($P < 0.05$, *t*-test); **NS, not significant compared with CDPP-based chemotherapy, VAC, FAM or FAMT and others.

Ulbright's enteric differentiation (Ulbright *et al.*, 1986). Recently, the International Society of Gynecological Pathologists proposed a new classification of YST of the ovary (International Society of Gynecological Pathologists, 1990). In the new classification, YST was subdivided into five groups: endodermal sinus tumour, polyvesicular vitelline variant, glandular variant, hepatoid variant and mixed type. The classification of YSTs was based on ES, PV, G and H subtypes. Hence, we classified YST according to the five subtypes (SAS Institute, 1985). There is interest in the clinical significance of the new classification of YST of the ovary. First, a study of the correlation between histological subtypes and prognosis was performed by Kurman and Norris (1976), who divided YSTs into five main histological subtypes. However, there was no correlation of these subtypes with prognosis. Nogales *et al.* (1978) reported that a pure polyvesicular vitelline subtype was associated with a better outcome for patients with testicular YST. However, there is currently no information to suggest that glandular and hepatoid subtypes are biologically different from other types of ovarian YST.

In the present study, multivariate analysis of all variables demonstrated that chemotherapeutic regimen, residual tumour size and FIGO stage are more important prognostic factors than the number of histological subtypes. Other investigators have reached similar conclusions (Kurman & Norris, 1976; Bradof *et al.*, 1982; Taylor *et al.*, 1985; Carlson *et al.*, 1983; Davis *et al.*, 1984). However, there have been few reports about the prognostic significance of histological

**Figure 5** Survival related to the number of histological subtypes in FIGO stage I patients with ovarian pure yolk sac tumours calculated by the Kaplan-Meier methods. **a**, One subtype (five patients); **b**, two subtypes (five patients); **c**, three or four subtypes (seven patients). Survival times were calculated from the time of primary therapy to death.

subtypes in FIGO stage I YST of the ovary (Julian *et al.*, 1980). Hence, in the present series, only stage I patients were further analysed to evaluate the prognostic significance of the various histological subtypes.

In patients with FIGO stage I disease, residual tumour size, FIGO stage I subclassification, age and chemotherapy were not important as prognostic factors. All patients with FIGO stage I disease had relatively complete surgery without residual tumour or with only ascites containing tumour cells. Surgical procedures, especially SO or BSO and TAH + BSO, had similar outcomes. Therefore, residual tumour size was ignored as a prognostic factor. In the subclassification of FIGO stage I, although stage Ic patients had worse prognosis than stage Ia or Ib patients, the survival difference between stage Ia or Ib and Ic was small. In addition, there was no bias concerning stage Ic between the group with one histological subtype and those with an admixture, since the disease stage in groups with one, two and three or four subtypes showed no significant differences ($P = 1.00$). Multivariate analysis showed that the subclassification of FIGO stage I had less significance than histological subtype. Einhorn, Gershenson and other investigators have reported that cisplatin-based regimens (PVB and BEP) is more effective than VAC in the treatment of ovarian germ cell tumours (Einhorn & Donohue, 1977; Julian *et al.*, 1980; Carlson *et al.*, 1983; Davis *et al.*, 1984). However, our data showed that chemotherapy regimen was not a significant predictor in FIGO stage I patients. In FIGO stage I, VAC produced a high proportion of cures, but in patients with metastatic disease the sustained remission rates of cisplatin-based chemotherapies are better than that of VAC (Pinkerton *et al.*, 1986; Williams *et al.*, 1989; Gershenson *et al.*, 1990). Therefore, our results of multivariate analysis in FIGO stage I patients are not consistent with other reports concerning chemotherapy for ovarian YST. In addition, there were no significant differences between groups with an admixture of histological subtypes and that with one subtype with respect to the number of patients treated with cisplatin-based chemotherapies, although patients with three or four subtypes had a better outcome than patients in other groups.

Next, age was considered as a possible prognostic factor. Some investigators have reported that there are no significant differences in prognosis between those younger and older than 20 years of age (Kurman & Norris, 1976; Kawai *et al.*, 1991). In the present study, multivariate analysis showed the same result as other reports. In addition, patients less than 10 years of age are known to have a worse prognosis, and in our series no FIGO stage I patient was less than 10 years old.

There were no statistically significant differences in the factors mentioned above among the three groups with different numbers of histological subtypes. However, there was a significant difference in alpha-fetoprotein between the stage I patients with two subtypes and those with three or four subtypes. However, both univariate and multivariate analysis showed no prognostic significance in relation to alpha-fetoprotein in this study. There are previous reports indicating that preoperative alpha-fetoprotein levels do not correlate with prognosis (Kawai *et al.*, 1991). Therefore, it can be considered that histological subtype is superior to other factors as a predictor in FIGO stage I YST of the ovary.

When we focused on the number of histopathological slides, there were variations in the numbers of slides examined for each histological subtype. The mean number of slides studied for tumours containing three or four subtypes was greater than that for tumours with one or two subtypes. There was a wide variation in the number of slides reviewed per patient. However, when statistical analysis was performed, there were no significant differences between tumours with one or two subtypes and those with three or four subtypes. However, when the group with three or four subtypes was separated for comparison with other groups, comparison of the group with one subtype and that with three subtypes showed a significant difference. Even though there were no significant differences in comparing the numbers of slides examined in the groups with three or four subtypes with other groups, it was thought that the number of subtypes might increase in some cases after histopathological examination of a large number of slides, especially in the tumours containing only one subtype.

Generally, it is agreed that well-differentiated germ cell tumours have better prognosis than undifferentiated ones. In comparing embryonic structures with neoplastic subtypes, YSTs containing three or four distinct subtypes may be better differentiated tumours that pure endodermal sinus tumours. If the concept were reasonable, it would be that patients with tumours containing three or four subtypes have a better prognosis than those with tumours containing one or two subtypes. Therefore, it could be clinically important to investigate the prognostic significance of histological subtype in stage I YST. The present study was a retrospective investigation, and may have a bias in the numbers of histopathological slides examined. We consider that a prospective study with identical pathological examinations is necessary to confirm the correlation between histological subtype and the clinical outcome in stage I pure yolk sac tumour of the ovary.

Although stage I pure yolk sac tumour generally has a good prognosis, the present study suggests that the different subtypes may have prognostic significance. In particular, endodermal sinus subtype alone is associated with a poor prognosis, which may have therapeutic implications.

The authors thank Hisayoshi Nakajima, Michiaki Yakushiji, Haruo Nishimura, Shigeaki Iwanaga, Shoji Jimi, Hisao Nakano, Naoki Tsukamoto, Akisuke Moriwaki, Masamichi Hiura, Michi Chiba, Yutaka Tomoda, Takeo Kanou, Mitihata Kawai, Nobuo Nakajima, Takahiko Sonodo, Shin Fukutomi, Katsuhiko Hasumi, Hideo Teshima, Masahiko Nishijima, Hiroyuki Kuramoto, Toshiko Jouboub, Satoshi Yajima, Shinji Satou, Tsuneo Namiki, Ijirou Morizuka, Kenichi Tanaka, Kouji Kanazawa, Syouji Kodama, Masatoshi Hashimoto, Ryuuichi Kudoh and Osamu Hayakawa for the supply of patient samples in this study. The authors thank Dr T.A. Tavassoli (Armed Forces Institue of Pathology, Washington DC 20306-6000) and Dr W. Gray (University of Otago, New Zealand) for help with preparation of the manuscript.

### References

BRADOF, J.E., HAKES, T.B., OCHOA, M. & GOLBEY, R. (1982). Germ cell malignancies of the ovary. Treatment with vinblastine, actinomycin D, bleomycin and cisplatin containing chemotherapy combinations. *Cancer*, **50**, 1070–1075.

CARLSON, R.W., SIKIC, B.I., TURBOW, M.M. & BALLON, S.C. (1983). Combination cisplatin, vinblastine and bleomycin chemotherapy (PVB) for malignant germ-cell tumors of the ovary. *J. Clin. Oncol.*, **1**, 645–651.

COX, D.R. (1972). Regression models and life tables. *J. R Stat. Soc.*, **34**, 187.

DAVIS, T.E., LOPRINZI, C.L. & BUCHLER, D.A. (1984). Combination chemotherapy with cisplatin, vinblastine and bleomycin for endodermal sinus tumor of the ovary. *Gynecol. Oncol.*, **19**, 46–52.

DUVAL, M. (1891). Le placenta de rongeurs. *J. Anat. Physiol.*, **27**, 515–612.

EINHORN, L.H. & DONOHUE, J. (1977). Cisdiamine dichloroplatinum, vinblastine, and bleomycin combination chemotherapy in disseminated testicular cancer. *Ann. Intern. Med.*, **87**, 293–298.

GERSHENSON, D.M., COPELAND, L.J., KAVANAGH, J.J., CANGIR, A., DEL JUNCO, G., SAUL, P.B., STRINGER, C.A., FREEDMAN, R.S., EDWARDS, C.L. & WHARTON, J.T. (1985). Treatment of malignant nondysgerminomatous germ cell tumors of the ovary with vincristine, dactinomycin and cyclophosphamide. *Cancer*, **56**, 2756–2761.

GERSHENSON, D.M., MORRIS, M., CANGIR, A., KAVANAGH, J.J., STRINGER, C.A. & EDWARDS, C.L. (1990). Treatment of malignant germ cell tumors of the ovary with bleomycin, etoposide and cisplatin (BEP). *J. Clin. Oncol.*, **8**, 715–720.

HUNTINGTON JR, R.W. & BULLOCK, W.K. (1970). Yolk sac tumors of the ovary. *Cancer*, **25**, 1357–1367.

HUNTINGTON JR, R.W., MORGENSTERN, N.L., SARGENT, J.A., GIEM, R.N., RICHARDS, A. & HANFORD, K. (1963). Germinal tumors exhibiting the endodermal sinus pattern of Teilum in young children. *Cancer*, **16**, 34–47.

INTERNATIONAL FEDERATION OF GYNECOLOGY AND OBSTETRICS (1988). *Annual Report on the Results of Treatment in Gynecological Cancer*, Vol. 20. Pettersson, F. (ed.), p. 111, Panorama Press: Stockholm.

INTERNATIONAL SOCIETY OF GYNECOLOGIC PATHOLOGY (1990). The new classification of ovarian tumours. In *Proceedings of International Academy of Pathology*, in Buenos Aires.

JIMERSON, G.K. & WOODRUFF, J.D. (1977). Ovarian extraembryonal teratoma. 1. Endodermal sinus tumor. *Am. J Obstet. Gynecol.*, **127**, 73–79.

JULIAN, C.G., BARRETT, J.M., RICHARDSON, R.L. & GRECO, F.A. (1980). Bleomycin, vinblastine, and cis-platinum in the treatment of advanced endodermal sinus tumor. *Obstet. Gynecol.*, **56**, 396–401.

KAPLAN, E. & MEIER, P. (1958). Non-parametric estimations from incomplete observations. *J. Am. Stat. Assoc.*, **53**, 457–482.

KAWAI, M., KANO, T., FURUHASHI, Y., MIZUNO, K., NAKASHIMA, N., HATTORI, S., KAZETO, S., IIDA, S., OHTA, M., ARII, Y. & TOMODA, Y. (1991). Prognostic factors in yolk sac tumors of the ovary. A clinicopathologic analysis of 29 cases. *Cancer*, **67**, 184–192.

KURMAN, R.J. & NORRIS, H.J. (1976). Endodermal sinus tumor of the ovary: a clinical and pathologic analysis of 71 cases. *Cancer*, **38**, 2404–2419.

NEUBECCKER, R.D. & BREEN, J.L. (1962). Embryonal carcinoma of the ovary. *Cancer*, **15**, 546–556.

NOGALES, F.F., MANTILLA, A., NOGALES-ORTIZ, F. & GALERA-DAVIDSON, H.L. (1978). Yolk sac tumors with pure and mixed polyvitelline subtypes. *Hum. Pathol.*, **9**, 553–566.

PINKERTON, C.R., PRITCHARD, J. & SPITZ, L. (1986). High complete response rate in children with advanced germ cell tumors using cisplatin-containing combination chemotherapy. *J. Clin. Oncol.*, **4**, 194–199.

PRAT, J., BHAN, A.K., DICKERSIN, G.R., ROBBOY, S.J. & SCULLY, R.E. (1982). Hepatoid yolk sac tumor of the ovary (endodermal sinus tumor with hepatoid differentiation): a light microscopic, ultrastructural and immunohistochemical study of seven cases. *Cancer*, **50**, 2355–2368.

SALAZAR, H., KANBOUR, A., TOBON, H. & GONZALEZ-ANGALO, A. (1974). Endoderm cell derivatives in embryonal carcinoma of ovary. An electron microscopic study of two cases (abstract). *Am. J. Pathol.*, **74**, 108a.

SAS INSTITUTE (1983). *SUGI Supplemental Library User's Guide*, pp. 267–294. SAS Institute: Cary, NC.

SAS INSTITUTE (1985). *SAS User's Guide: Statistics*, version 5, pp. 529–557. SAS Institute: Cary, NC.

SMALES, E. & PECKMAN, M.J. (1987). Chemotherapy of germ-cell ovarian tumours: first-line treatment with etoposide, bleomycin and cisplatin or carboplatin. *Eur. J. Cancer Clin. Oncol.*, **23**, 469–474.

TALERMAN, A., HAIJE, W.G. & BAGGERMAN, L. (1978). Serum alpha-fetoprotein in diagnosis and management of endodermal sinus (yolk sac tumor) and mixed germ cell tumor of the ovary. *Cancer*, **41**, 272–278.

TAYLOR, M.H., DEPETRILLO, A.D. & TURNER, A.R. (1985). Vinblastine, bleomycin and cisplatin in malignant germ cell tumors of the ovary. *Cancer*, **56**, 1341–1349.

TEILUM, G. (1959). Endodermal sinus tumors of the ovary and testis. Comparative morphogenesis of the so-called mesonephroma ovarii (Schiller) and extraembrynic (yolk sac-allantoic) structures of the rat's placenta. *Cancer*, **12**, 1092–1105.

TEILUM, G. (1965). Classification of endodermal sinus tumour (mesoblastoma vitellinum) and so-called embryonal carcinoma of the ovary. *Acta Pathol. Microbiol. Scand.*, **64**, 407–429.

TEILUM, G. (1968). Tumours of germinal origin. In *Ovarian Cancer*,UICC Monograph Series, Vol. 11, Gentil, F. & Junqueira, A.C. (eds) pp. 58–73. Springer: Berlin.

TEILUM, G. (1976). *Special Tumors of the Ovary and Testis. Comparative Histology and Identification*, 2nd edn. Munksgaard: Copenhagen.

ULBRIGHT, T.M., ROTH, L.M. & BRODHECKER, C.A. (1986). Yolk sac differentiation in germ cell tumors. A morphologic study of 50 cases with emphasis on hepatic, enteric and parietal yolk sac features. *Am. J. Surg. Pathol.*, **10**, 151–164.

WILLIAMS, S.D., BLESSING, J., SLAYTOR, R., HOMESLEY, H. & PHOTOPOLUS, G. (1989). Ovarian germ cell tumors: adjuvant trials of the Gynecologic Oncology Group (abstract). *Proc. Am. Soc. Clin. Oncol.*, **8**, 150.